# Muscle strength, muscle power and body composition in college-aged young women and men with Generalized Joint Hypermobility

**DOI:** 10.1371/journal.pone.0236266

**Published:** 2020-07-29

**Authors:** Paulina Ewertowska, Zbigniew Trzaskoma, Dominik Sitarski, Bartłomiej Gromuł, Ireneusz Haponiuk, Dariusz Czaprowski

**Affiliations:** 1 Department of Rehabilitation and Kinesiology, Gdansk University of Physical Education and Sport, Gdansk, Poland; 2 Faculty of Rehabilitation, Józef Piłsudski University of Physical Education, Warsaw, Poland; 3 Department of Physiotherapy, Józef Rusiecki University College, Olsztyn, Poland; 4 Department of Rehabilitation, Poznan University of Medical Sciences, Poznan, Poland; University of Extremadura, SPAIN

## Abstract

The aim of this study was an evaluation of the musculoskeletal system in women and men with Generalized Joint Hypermobility (GJH). The study included 87 participants– 40 with Generalized Joint Hypermobility (aged 21.2 ±1.8 years) and 47 (aged 21.0 ±1.3 years) in the control group (CG). The study included the Beighton score, the measurements of body composition, muscle flexibility (Straight Leg Raise test, Popliteal Angle test, Modified Thomas Test), and the measurements of muscle strength and muscle power. T-test and Mann-Whitney U Test were applied to assess the differences between independent groups. The study showed that there were no significant differences (p>.05) in the assessed body composition and the muscle flexibility between both women and men with GJH and the participants in the CG. Under isokinetic conditions for the non-dominant lower extremity, men from the CG received significantly higher (p = .02) flexion peak torque at 180°/s angular velocity. Women from the CG received a statistically significantly lower (p = .04) F/E ratio at 180°/s velocity. Under isometric conditions for both women and men with GJH, there were no statistically significant differences (p>.05) in the maximum torques in knee extension and flexion compared to the CG. For women and men with GJH, the maximum power in the lower extremities and jumping ability were not significantly different (p>.05) compared to the CG participants. The body composition, muscle flexibility, muscle strength, and muscle power of adults with Generalized Joint Hypermobility did not differ compared to healthy participants. The fact that there are no differences does not exclude the efficacy of strength training in increasing levels of muscle strength and its impact on body posture and proprioception or coordination.

## Introduction

Generalized Joint Hypermobility (GJH) is defined as an increased range of motion in large and small joints, taking into account age, gender, and ethnicity [1–4]. The prevalence of GJH is reported to be from 10% to 20% [5]. It occurs three times more often in women than in men [6,7]. GJH is characterized by increased mobility of the peripheral and interstitial joints, tenderness of the articular capsules, and consequently, a decreased mechanical and stabilizing quality of the joints [4]. GJH is a musculoskeletal disorder without any other systemic diseases in contrast to the Benign Joint Hypermobility Syndrome (BJHS). In the BJHS case, chronic pain is characteristic [4,8]. The pathophysiology of the GJH lays in the disproportion between the increased quantity of type I collagen relative to type III [9]. Researchers confirm the coexistence of increased mobility in joints with spinal and peripheral joint pain and postural disorders such as idiopathic scoliosis, flat feet, impaired proprioception, and coordination [10–14]. In children with hypermobility, a lower level of physical activity, and consequently reduced muscle strength and physical performance can be observed [14].

GJH diagnosis is based on clinical symptom analysis [4]. The most commonly used diagnostic method is the Beighton 9-point test [1,15]. Other methods assessing GJH are the Carter and Wilkinson test, the Marshall test, and the Bulbena scale [12].

There is no causal treatment of joint hypermobility [16]. The therapy should be based on the individual needs of patients, and clinical expertise [16,17]. A physiotherapist's knowledge of GJH tends to be relatively low. Inadequate physiotherapy including flexibility exercises or aggressive manual techniques in hypermobile joints can cause a patient’s health condition to deteriorate. Therefore, it seems necessary to include specifications for the GJH assessment of participants with GJH in standard physiotherapeutic diagnostics [10,18]. According to Keer and Grahame [18], physiotherapy is the pillar of treatment in the musculoskeletal consequences for GJH. Specific training can improve the condition of the muscular system as a dynamic stabilizer [19]. Existing published analysis suggests that there are no evidence-based strategies in the treatment of GJH. Case studies confirm the effectiveness of physiotherapeutic interventions in the treatment of GJH but leave many ambiguities [17].

The aim of this study was an assessment of the musculoskeletal system of women and men with GJH. It included the measurements of body composition, pelvis-hip complex muscle flexibility, muscle strength, muscle power, and jumping ability.

## Materials and methods

### Participants

The recruitment process began with the announcement of a research project at the university. The next step was the informing meeting about the aim and protocol of the study. Then one researcher conducted the measurements of hypermobility, muscle flexibility, strength, and power. The study was conducted on 91 participants willing to participate in the research project. During the first meeting, all participants were informed about the aim and protocol of the study and the option to discontinue their participation at any time.

The exclusion criteria were: Benign Joint Hypermobility Syndrome (the Brighton criteria), knee varus or valgus (measured Q angle), orthopedic, rheumatological, neurological or genetic disorders, pain in lower extremities and lower back pain (interview information). Three participants were excluded because of chronic pain and one due to knee arthroscopy.

Ultimately, the research included 87 participants– 40 with GJH (25 women and 15 men) and 47 (28 women and 19 men) classified as the CG.

Before the study, the number of participants was calculated. 17 participants were recommended for each group. The analysis was conducted with 95% of confidence interval (α = 0.05), power of test 0.80, and sigma 0.15 N m/kg of body weight. The sigma was specified after the preliminary study.

In this study, all musculoskeletal performance tests were characterized by reliability and repeatability [4,20–25]. The participants did not know to which group–with GJH or control the participants belonged.

The local ethics committee (Senacka Komisja Etyki Badań Naukowych–SKE01-2/2014) consent was obtained prior to the study. Written participants’ consent was obtained.

### Assessment of generalized joint hypermobility

The assessment of GJH was carried out using the 9-point Beighton score [20]. It included: a) abduction of the thumb to the forearm, b) knee hyperextension above 10 degrees, c) extension of the metacarpophalangeal joint in the 5^th^ finger above 90 degrees, d) elbow hyperextension above 10 degrees and e) placing flat hands on the floor. Each positive test would score 1 point. Females with ≥5 and males with ≥4 scores in the Beighton score were included in the GJH groups [26,27]. The single-blind test was applied. The assessment was carried out at the goniometer (MSD Europe Bvba, North America).

### Assessment of body composition

The measurement of body composition was carried out at Tanita–BC 418 MA (Tanita Corporation, Japan). The assessment included the quantity of fat mass (%, kg), fat-free mass (kg), and total body water (kg). Weight of clothing [28] was assumed as 0.5 kg.

### Assessment of pelvic-hip complex muscle flexibility

The measurement of hamstring flexibility was carried out during the Straight Leg Raise Test (SLR) and the Popliteal Angle Test (PA) [20,29]. One-Joint Hip Flexors (O-JHF) and Two-Joint Hip Flexors (T-JHF) flexibility were conducted during the modified Thomas Test. The assessment was performed by two researchers. The first researcher (R1) measured the angle of the joint of the lower extremity with AMI digital inclinometer (OPIW, Poland), which was reset into a horizontal position before each measurement. The second researcher (R2) carried out the test and stabilized the participant’s position. The mean value of the three measurements was taken into statistical analysis [20]. The measurements were carried out for both lower extremities.

### Straight Leg Raise Test

The participant lay in a supine position with the target lower extremity relaxed. The second lower extremity was positioned behind the table for the lumbar spine and pelvis stabilization. The R2 controlled the position of the participant and raised their lower extremity with the knee extended to the first resistance of the hamstring. The R1 assessed the range of hip flexion with an inclinometer, which was placed 10 cm proximally to the patella [20].

### Popliteal Angle Test

The participant lay in a supine position with the hip flexed to 90°. He stabilized his thigh position with both hands. The R2 controlled this position with a goniometer, which was set over his greater trochanter. Its stationary arm was located along the trunk (parallel to the table) and the mobile arm along the femur. In this position, the participant had to straighten his knee. The R1 assessed the popliteal angle with an inclinometer, which was placed 10 cm below the tibial tuberosity. The untested lower extremity was extended and relaxed [20].

### Modified Thomas Test

A Modified Thomas Test was used to assess the flexibility of O-JHF and T-JHF [20]. The participant lay in a supine position with their pelvis close to the edge of the table. The untested lower extremity was flexed at the hip and knee joint to stabilize the pelvis and the lumbar spine. The participant held this position with their hands. The R2 controlled and stabilized the participant’s position. The tested lower extremity was placed in a relaxed position behind the table. The R1 assessed the range of hip extension or flexion with an inclinometer, which was placed 10 cm proximally to the patella.

During the T-JHF test, the participant and R2 were in the same position. The R1 assessed the range of knee flexion with an inclinometer, which was placed 10 cm below the tibial tuberosity [20].

### Assessment of muscle strength

A priori, the study included testing the level of dominance in the functional lower extremities by kicking the ball to the target [30,31]. The lower extremity which kicked the ball was defined as the dominant leg [30,31].

The isometric and isokinetic relative peak torque for the knee flexors and extensors was measured using the Biodex System Pro 3 (Biodex Medical Systems, Shirley, NY, USA) with Biodex Advantage Software v.4.0. The participant was placed in a sitting position on a chair with a backrest. The thorax, hip, and calf were stabilized with straps [31]. The anatomical axis of rotation of the knee joint was consistent with the dynamometer axis. The knee was kept at a 90° flexion, the hip in neutral rotation and abduction, and the foot was relaxed. The Biodex was calibrated before each measurement [31].

Under isometric conditions, the same test for knee flexion (30° knee angle) and for knee extension (70° knee angle) [32,33] was repeated 3 times for each. The participant was instructed to flex/extend their knee and to exert a maximum contraction on the command “Go” of the researcher.

Under isokinetic conditions, the participant undertook the same test 5 times for the knee flexion/extension with 60°/s angular velocity and 10 times for movement with 180°/s [31,34,35]. The range of motion was 0–90° degrees for a knee joint [31,36,37]. A 5-second rest was given between each trial to avoid fatigue. A 3-minute rest was provided after testing the dominant extremity before testing the non- dominant extremity [37]. In this study, the peak torque for each muscle group (Nm/kg) and Flexors/Extensors (F/E) ratio was analyzed. A 5-minute warm-up was carried out on a cycloergometer with 65–85% maximal heart rate before all measurements of strength were made [38].

The measurements of muscle strength under isokinetic conditions were conducted by many researchers. The recommended angular velocities for assessment of hypermobile knee joints are 60°/s, 180°/s, and 240°/s [31,34,35]. The measurement protocol was recommended by the producer of Biodex System PRO3.

### Assessment of muscle power and jumping ability

A 5-minute rest was provided after testing muscle strength before testing muscle power. The measurements of maximal muscle power of lower extremities and jumping ability (height of the center of mass) were carried out during Akimbo Countermovement Jump (ACMJ) on a local made PLA2–4P force platform (JBA, Poland) with MVJ software v 3.4 [39,40]. The participant was placed in a standing position with their hands on hips. This position was adopted during all phases of the jump. The participant repeated the maximal vertical jump 3 times. The lowering of the center of mass during the jump was dependent on the individual technique of the participant [34]. A 5-second rest was provided between each jump. Relative values of maximal power (W/kg) and the height of the jump were analyzed. (cm) [34].

The entire testing (assessment of GJH, body composition, muscle flexibility, strength, and power) took 45 minutes.

### Statistical analysis

The statistical analysis was performed using Statistica 13.1 (StatSoft, Poland). The descriptive statistics were calculated separately for the group with GJH and the CG. Normal distribution was assessed with the use of the Shapiro-Wilk Test. T-test and Mann-Whitney U Test were applied to assess the differences between study and control groups. The value p = .05 was adopted as the level of significance with a 95% confidence interval. One week before the study a preliminary test was conducted. It included 13 participants who were later included in the study group. This test allowed to verify the adopted method of measurements.

## Results

### Preliminary study

The preliminary study was conducted according to the same rules as the main research project. The preliminary study showed that the methods applied were well received by the participants. The measurements did not cause any fatigue, which could have a negative impact on the results. The biggest differences between the GJH and the CG was observed in the dominant lower extremity during SLR Test (76.3° vs 67.7°) and PA test (79.7° vs 64.1°) and during knee extension (3.03 vs 2.64 Nm/kg) in isometric conditions.

### Participants

The participants aged 19–25 years (21.1±1.6). A list of detailed characteristics is presented in Table 1. There were no significant differences between people with GJH and the CG in terms of age, weight, height, body mass index (BMI), and declared level of physical activity. All participants performed recreational aerobic physical activity.

**Table 1:** Characteristics of women and men with GJH and from the CG.

	Females n = 53			Males n = 34		
	GJH	CG	p value	GJH	CG	p value
	n = 25	n = 28		n = 15	n = 19	
	Me (IQR)	Me (IQR)		Me (IQR)	Me (IQR)	
**Age (years)**	21.0 (20.0–23.0)	21.0 (19.0–22.0)	.68	21.0 (20.0–21.0)	21.0 (20.0–22.5)	.06
**Weight (kg)**	60.1 (54.8–67.9)	61.2 (54.5–70.1)	.55	80.0 (75.0–87.0)	78.0 (72.9–82.7)	.42
**Height (m)**	1.7 (1.6–1.7)	1.7 (1.6–1.7)	.13	1.8 (1.8–1.9)	1.8 (1.8–1.9)	.49
**BMI (kg/m**^**2**^**)**	21.9 (19.8–24.6)	21.8 (19.7–25.8)	.72	24.6 (22.4–25.8)	24.1 (20.9–25.3)	.31
**Physical activity (h/week)**	3.0 (2.0–6.0)	4.0 (2.0–6.0)	.27	6.0 (5.0–7.5)	6.5 (4.0–11.5)	.24

Abbreviations: GJH–Generalized Joint Hypermobility, CG–Control Group, Me–Median, IQR–Interquartile Range.

### Assessment of generalized joint hypermobility

The average result in the 9-point Beighton score in the GJH group was 6.3±1.4 (Me 6.0, IQR 5–7) for women and 4.9±1.1 (Me 4.5, IQR 4–6) for men. Women in the CG had an average score of 1.4±1.2 (Me 1.0, IQR 0–2) and men 0.5±0.7 (Me 0.0, IQR 0–1). The minimum and maximum scores for women with GJH were 5 and 9, and for men 4 and 7 points, respectively. In the CG, the minimum and maximum scores for women were 0 and 4, and for men 0 and 2 points, respectively.

### Assessment of body composition

The body composition analysis showed that women with GJH achieved similar scores in fat mass, fat-free mass, and total body water compared to the CG. There were no significant differences between men with GJH and from the CG in terms of body composition (Table 2).

**Table 2:** The comparison of body compositions between females and males with and without generalized joint hypermobility.

	Females n = 53			Males n = 34		
	GJH	CG	p value	GJH	CG	p value
	n = 25	n = 28		n = 15	n = 19	
	Mean (SD)	Mean (SD)		Mean (SD)	Mean (SD)	
**Fat mass (%)**	28.4 (5.8)	29.2 (7.1)	.68	14.2 (3.6)	14.6 (5.7)	.82
**Fat mass (kg)**	17.9 (6.0)	19.8 (8.7)	.41	11.6 (3.8)	11.5 (4.7)	.98
**Fat-free mass (kg)**	43.6 (3.2)	45.3 (5.9)	.56	69.2 (7.0)	66.7 (6.1)	.37
**Total body water (kg)**	31.9 (2.4)	33.1 (4.3)	.57	50.7 (5.1)	48.8 (4.5)	.36

Abbreviations: GJH–Generalized Joint Hypermobility, CG–Control Group, SD–Standard Deviation.

### Assessment of pelvic-hip complex muscle flexibility

Women with GJH and from the CG got similar results of the flexibility of hamstring (SLR Test and PA Test), One and Two-Joint Hip Flexors. There were also no significant differences (p>.05) between men with GJH and the CG in respect of muscle flexibility (Table 3).

**Table 3:** The comparison of muscle flexibility tests between females and males with and without generalized joint hypermobility.

	Females n = 53			Males n = 34		
	GJH	CG	p value	GJH	CG	p value
	n = 25	n = 28		n = 15	n = 19	
Lower extremity	Mean (SD)	Mean (SD)		Mean (SD)	Mean (SD)	
**Dominant**						
**SLR (°)**	65.5 (9.7)	65.8 (8.9)	.91	57.9 (12.9)	56.7 (9.7)	.81
**PA (°)**	67.8 (12.0)	66.0 (8.8)	.55	64.1 (13.7)	61.2 (9.2)	.81
**O-JHF (°)**	17.5 (8.0)	16.1 (7.5)	.46	11.1 (15.3)	4.2 (16.4)	.27
**T-JHF (°)**	78.4 (7.8)	78.1 (9.2)	.88	68.6 (9.7)	73.9 (9.1)	.16
**Non-dominant**						
**SLR (°)**	63.9 (9.6)	63.8 (10.5)	.96	55.6 (12.9)	57.1 (9.0)	.74
**PA (°)**	67.9 (10.9)	66.6 (8.4)	.61	63.6 (15.3)	58.7 (11.7)	.54
**O-JHF (°)**	18.9 (9.6)	16.7 (8.9)	.26	13.1 (13.8)	5.6 (14.5)	.18
**T-JHF (°)**	78.5 (8.1)	77.3 (8.7)	.61	65.9 (14.1)	72.5 (10.4)	.18

Abbreviations: GJH–Generalized Joint Hypermobility, CG–Control Group, SD–Standard Deviation, SLR–Straight Leg Raise test, PA–Popliteal Angle test, O-JHF–One-Joint Hip Flexors test; T-JHF–Two-Joint Hip Flexors test.

### Assessment of muscle strength

Under isokinetic conditions, women with GJH obtained a similar relative value of muscle peak torque during knee flexion and extension, both at 60°/s and 180°/s angular velocity compared to the CG. At 180°/s velocity, women with GJH obtained higher values F/E ratio for the non-dominant than for the dominant lower extremity, respectively 0.55 and 0.50. These values for women from the CG were equal to 0.48 for both extremities. Women from the CG received a statistically significantly lower (p = .04) F/E ratio at 180°/s velocity for the non-dominant lower extremity (Table 4).

**Table 4:** The comparison of peak torque under isokinetic conditions obtained by females and males with and without generalized joint hypermobility.

Peak torque/Body weight (Nm/kg)		Females n = 53			Males n = 34		
		GJH	CG	p value	GJH	CG	p value
		N = 25	n = 28		n = 15	n = 19	
Lower extremity		Mean (SD)	Mean (SD)		Mean (SD)	Mean (SD)	
**Dominant**							
**Flexion**	**60°/s**	0.87 (0.19)	0.90 (0.23)	.57	1.17 (0.24)	1.28 (0.32)	.39
	**180°/s**	0.64 (0.18)	0.63 (0.21)	.97	0.82 (0.19)	0.91 (0.23)	.31
**Extension**	**60°/s**	2.13 (0.31)	2.05 (0.43)	.53	2.55 (0.39)	2.63 (0.37)	.54
	**180°/s**	1.29 (0.21)	1.29 (0.33)	.46	1.55 (0.35)	1.68 (0.15)	.23
**F/E (-)**	**60°/s**	0.41 (0.07)	0.44 (0.07)	.29	0.47 (0.13)	0.48 (0.10)	.74
	**180°/s**	0.50 (0.12)	0.48 (0.10)	.57	0.54 (0.09)	0.57 (0.13)	.39
**Non-dominant**							
**Flexion**	**60°/s**	0.85 (0.17)	0.86 (0.21)	.87	1.07 (0.24)	1.21 (0.30)	.12
	**180°/s**	0.66 (0.15)	0.60 (0.24)	.16	0.73 (0.18)	0.93 (0.23)	.04*
**Extension**	**60°/s**	2.00 (0.28)	2.05 (0.44)	.79	2.15 (0.63)	2.59 (0.42)	.02*
	**180°/s**	1.23 (0.18)	1.25 (0.33)	.36	1.39 (0.37)	1.65 (0.33)	.03*
**F/E (-)**	**60°/s**	0.43 (0.09)	0.42 (0.06)	.74	0.52 (0.11)	0.47 (0.09)	.16
	**180°/s**	0.55 (0.11)	0.48 (0.12)	.04*	0.54 (0.07)	0.57 (0.11)	.40

Abbreviations: GJH–Generalized Joint Hypermobility, CG–Control Group, SD–Standard Deviation, F/E–Flexion/Extension ratio

* Statistically significant differences.

Men with GJH achieved similar results during extension and flexion of the dominant lower extremity compared to the CG. For the non-dominant lower extremity, men with GJH received significantly lower (p = .04) flexion peak torque at 180°/s angular velocity and extension peak torque at 60°/s and 180°/s, respectively p = .02 and p = .03. Both in the studied group and the CG, men obtained the same F/E ratio during 180°/s angular velocity (Table 4).

Under isometric conditions, women with GJH obtained a similar relative peak torque during flexion and extension.

Men with GJH and from the CG received no different peak torques during extension (dominant extremity) and F/E ratio (non-dominant extremity). The remaining results of strength were similar in the study group than in the CG (Table 5).

**Table 5:** The comparison of peak torque under isometric conditions obtained by females and males with and without generalized joint hypermobility.

Peak torque/Body weight (Nm/kg)	Females n = 53			Males n = 34		
	GJH	CG	p value	GJH	CG	p value
	n = 25	n = 28		n = 15	n = 19	
Lower extremity	Mean (SD)	Mean (SD)		Mean (SD)	Mean (SD)	
**Dominant**						
**Flexion 30°**	1.30 (0.22)	1.38 (0.24)	.25	1.85 (0.20)	1.93 (0.26)	.36
**Extension 70°**	2.28 (0.41)	2.32 (0.55)	.76	3.02 (0.47)	3.02 (0.48)	.98
**F/E (-)**	0.58 (0.08)	0.61 (0.11)	.25	0.62 (0.08)	0.65 (0.07)	.37
**Non-dominant**						
**Flexion 30°**	1.20 (0.20)	1.28 (0.22)	.19	1.64 (0.26)	1.82 (0.29)	.08
**Extension 70°**	2.11 (0.46)	2.32 (0.48)	.16	2.69 (0.79)	2.82 (0.38)	.09
**F/E (-)**	0.59 (0.13)	0.56 (0.11)	.56	0.65 (0.18)	0.65 (0.08)	.28

Abbreviations: GJH–Generalized Joint Hypermobility, CG–Control Group, SD–Standard Deviation, F/E–Flexion/Extension ratio.

### Assessment of muscle power and jumping ability

Women with GJH obtained similar muscle power for the lower extremities and height of jump compared to the CG during the Akimbo Countermovement Jump. In the same test, men with GJH obtained a similar score for jumping ability and for muscle power in comparison with the CG (Table 6).

**Table 6:** The comparison of maximal muscle power and jumping ability obtained by females and males with and without generalized joint hypermobility.

	Females n = 53			Males n = 34		
	GJH	CG	p value	GJH	CG	p value
	n = 25	n = 28		N = 15	n = 19	
	Mean (SD)	Mean (SD)		Mean (SD)	Mean (SD)	
**Maximal Power (W/kg)**	16.6 (3.8)	17.1 (4.2)	.66	23.1 (3.6)	22.8 (4.7)	.85
**Height of jump (cm)**	25.3 (2.9)	25.9 (3.9)	.52	34.3 (5.8)	35.3 (5.4)	.64

Abbreviations: GJH–Generalized Joint Hypermobility, CG–Control Group, SD–Standard Deviation.

## Discussion

The aim of the study was an evaluation of the musculoskeletal system of women and men with GJH.

### Body composition

The results of the body compositions were not significantly different for people with GJH and from the CG. We were not able to find in the literature available publications containing detailed results of the body compositions for adults with GJH. Engelbert et al. [41] showed that 12-year-old boys with GJH had higher body mass and BMI compared to the CG. Sohrbeck-Nøhra’s study is confirmed their results [42].

### Pelvic-hip complex muscle flexibility

Women and men with GJH obtained similar values of hamstring flexibility (SLR and PA tests) and for One- and Two-Joint Hip Flexors (O-JHF, T-JHF) compared to the control groups. Czaprowski et al. [20] did not notice any significant differences between girls and boys aged 10–13 with GJH in comparison with the control groups. Their study included the modified Finger-To-Floor test (FTF) and the Lateral Trunk Flexion test (LTF). Czaprowski et al. [20] claimed that the clinical examination of the pelvic-hip complex muscle and trunk flexibility was insufficient in diagnosing GJH in children and the Beighton score should be considered a standard element of physiotherapeutic examination of the musculoskeletal system.

### Muscle strength

The measurements of muscle strength in people with GJH have been the participant of much research [17,34,43,44,45], but the results presented do not allow a clear determination of the effect of joint hypermobility on the level of this feature. According to Jindal et al. [45], this is due to the lack of consideration in the interpretation of muscle strength measurements e.g. human race population, sex, age, functional dominance of extremities, or a variety of muscle groups to be measured [45].

Under isokinetic conditions, women with GJH obtained similar results of peak torque compared to the CG. Moreover, women from the CG received significantly lower F/E ratio (non-dominant extremity) compared to the remaining groups. This was due to the achievement of low values of peak torque during the extension of the knee joint. In men, all results for peak torque were similar to the CG. Only the value of strength during flexion at 180°/s and extension at 60°/s and 180°/s angular velocity was significantly lower for men with GJH. Liaghat et al. [46] claimed that higher velocities such as 180°/s have been defined as corresponding to muscle power (the ability to generate as much force and as fast as possible) and under 120°/s as corresponding to strength (the amount of force muscles can exert against an external load) [46]. Our study showed that it was possible that men with GJH had greater problems with muscle power than with muscle strength. However, the results of Akimbo Countermovement Jump did not confirm this tendency. These results were unlike that found by Liaghat et al. [46], who observed lower peak torque at 60°/s angular velocity, representing strength deficit in young competitive swimmers with GJH.

Juul-Kristensen et al. [34] applied a similar methodology in the examination of people with GJH. They used the Kinetic Communicator. They did not observe many differences in the relative strength of participants with GJH compared to the CG. However, in the case of girls and women with GJH, a significantly lower peak torque for the extension of the knee joint under isokinetic conditions was observed [34]. These results are a partial confirmation of our study, where men with GJH have seen a decrease in strength during knee flexion and extension for non-dominant lower extremity.

This study showed that women and men with GJH had a tendency to lower value peak torque under isometric conditions compared to the CG, but the differences were not statistically significant. Similar results were presented by Jensen et al. [43] and Mebes et al. [47]. Engelbert et al. [17] and Junge et al. [44] did not observe any differences in strength in children with GJH under isometric conditions compared to the CG. Juul-Kristensen et al. [34] and Massy-Westropp and Toubi [48] did not find significant differences in hand-grip between people with joint hypermobility and healthy participants. Yazgan and Duymaz [49] reported a lower strength for hand-grip in women with GJH than in the CG, but there were no significant differences in muscle strength in the lower extremity.

In this study participants with GJH had a tendency to manifest lower peak torque, especially under isometric conditions. The International Classification of Functioning, Disability and Health–ICF confirms the existence of lower muscle strength under isokinetic and isometric conditions [50], which occurs in Benign Joint Hypermobility Syndrome (BJHS) [35,51].

Jindal et al. [45] carried out their study using the Primus RS Isokinetic Dynamometer. They observed that men with GJH obtained significantly lower strength during elbow extension and knee extension (dominant extremity) compared to the CG. There were no differences between women with GJH and from CG [45]. This is a confirmation of our results, despite another angle of the knee joint during measurements (90° in Jindal’s et al. and 70° in our study).

Jensen et al. [43] showed that children and adults with GJH obtained similar results of muscle strength during knee extension and flexion and for F/E ratio compared to the control groups. In adults with GJH, the value of peak torque during knee extension was higher than in this study. However, during flexion, it was lower. This caused a distinct difference in the F/E ratio, which in Jensen et al. [43] was 0.38, and 0.6 (-) in our study, respectively. The differences could be caused by the participant groups’ different age profiles and the knee angle of equal to 100° [43]. According to Jensen et al. [43], low muscle strength during flexion and high during extension may indicate that there is a compensatory strategy to increase the stability of the knee joint.

Scheper et al. [16] claimed that the reduction of muscle strength is related to joint instability, worse proprioception, and pain. One of the criteria for differentiating people with GJH from people with Hypermobility Syndrome (HS) or Benign Hypermobility Syndrome (BHS) is back pain and pain in the peripheral joints [4,11,13]. In literature, there is evidence of a significant reduction in muscle strength in people with joint pain [34,35,51]. It should then be assumed as the inclusion and exclusion criteria from the study.

Sahin et al. [35] in researching the BIODEX System PRO 3 observed a reduction of peak torque during knee extension. They reported a decreased level of physical activity caused by pain [35] as a reason for this. According to Fatoye et al. [51], children with Joint Hypermobility Syndrome (JHS) manifested a reduction of peak torque during knee extension and flexion under isokinetic conditions [51]. Scheper et al. [52] claimed that people with GJH can modify their behavior, avoid dynamic activities, and refrain from erratic movement in order to prevent musculoskeletal problems. Remvig et al. [53] were unable to demonstrate any increased prevalence of joint pain, reduced motor competence, or reduced physical activity in school children with GJH or BJHS. But Sohrbeck-Nøhr et al. [42] claimed that it is possible to link between GJH with joint pain in the adolescent population. Chronic pain is common in the BJHS [4,8].

### Muscle power and jumping ability

The Akimbo Countermovement Jump (ACMJ) is a reliable method for the measurement of muscle power in lower extremities. It is used in the assessment of top-class athletes [54].

In this study, women and men with GJH achieved similar results for jumping ability (height of jump) and relative power compared to the control groups. Juul-Kristensen et al. [34] have not observed any differences between adults with GJH and from the CG during ACMJ on AMTI platform but children with GJH were found to have significantly higher values of jumping ability in comparison with the CG. Remvig et al. [53] used the Abalakov Test to assess the height of the jump. In children with GJH, there was no significant change in the jumping ability measured using the Beighton score [53]. The Sohrbeck-Nøhr et al. [42] study did not show any significant differences in children achieving 4, 5, and 6 points in the Beighton score.

### Limitations and clinical relevance

The study included measurements of body composition and muscle flexibility as factors affecting muscle strength and muscle power. The limitation of this study was the fact that the studies did not take into account the measurements of upper extremities and trunk muscle strength. The measurements did not include the assessment of all muscles surrounding the joints evaluated in the Beighton score. The aim of physiotherapy in people with GJH is the elimination of postural disorders and impaired proprioception or coordination [10–14]. The authors recognized that the function of the upper extremities had little impact on the occurrence of these disorders.

The results of this study did not show many differences in muscle strength between the GJH group and the CG. A decreased muscle strength in people with GJH could suggest that an increase in the level of this feature should be the therapeutic goal. That there are no differences does not exclude the efficacy of strength training in increasing levels of muscle strength in order to improve the function of the musculoskeletal system in people with GJH beyond that found in healthy subjects. This issue would require further research to examine the impact of strength training on body posture and the shaping of proprioception or coordination.

## Conclusions

1. The comprehensive diagnosis, which included the assessment of body components, muscle flexibility, muscle strength, power, and jumping ability did not show many differences between young adults with Generalized Joint Hypermobility and healthy people.

2. Under isometric conditions, the peak torque does not differ in young adults with Generalized Joint Hypermobility compared to the CG.

3. Healthy men obtained a higher peak torque for the non-dominant extremity under isokinetic conditions compared to young adults with Generalized Joint Hypermobility.

## Supporting information

S1 Table Characteristics of women and men with GJH and from the CG. (DOC)

S2 Table The comparison of body compositions between females and males with and without Generalized Join Hypermobility. (DOC)

S3 Table The comparison of muscle flexibility tests between females and males with and without Generalized Joint Hypermobility. (DOC)

S4 Table The comparison of peak torque under isokinetic conditions obtained by females and males with and without Generalized Joint Hypermobility. (DOC)

S5 Table The comparison of peak torque under isometric conditions obtained by females and males with and without Generalized Joint Hypermobility. (DOC)

S6 Table The comparison of maximal muscle power and jumping ability obtained by females and males with and without Generalized Joint Hypermobility. (DOC)

## References

[1] Malfait F, Hakim AJ, De Papee A, Grahame R. The genetic of the joint hypermobility syndromes. Rheumatology. 2006;45:502–507 10.1093/rheumatology/kei268 16418200

[2] Juul-Kristensen B, Kristensen JH, Frausing B, Jensen DV, Rogind H, Remvig L. Motor Competence and Physical Activity in 8-Year-Old School Children With Generalized Joint Hypermobility. Pediatrics. 2014;124(5):1380–1387 10.1542/peds.2009-0294 19822597

[3] Beighton P, Grahame R, Bird H. Musculoskeletal Features of Hypermobility and Their Management. Hypermobility of Joints. 4th ed London: Springer 2012;65–99b

[4] Juul-Kristensen B, Schmedling K, Rombaut L, Lund H, Engelbert RHH. Measurement properties of clinical assessment methods for classifying generalized joint hypermobility—A systematic review. American Journal of Medical Genetics Part C: Seminars in Medical Genetics. 2017;175(1):116–147 10.1002/ajmg.c.31540 28306223

[5] Hakim A, Grahame R. Joint hypermobility. Best Practice & Research: Clinical Rheumatology. 2003;17:989–1004, 10.1016/j.berh.2003.08.001 15123047

[6] Russek LN, Errico DM. Prevalence, injury rate and, symptom frequency in generalized joint laxity and joint hypermobility syndrome in a "healthy" college population. Clinical Rheumatology. 2016;35(4):1029–39 10.1007/s10067-015-2951-9 25930211

[7] Clinch J, Deere K, Sayers A, et al Epidemiology of generalized joint laxity (hypermobility) in fourteen-year-old children from the UK: a population-based evaluation. Arthritis Rheumatology. 2011;63(9):2819–27 10.1002/art.30435 PMC3164233 21547894

[8] Kulik O, Gębska M. Comparison of Beighton score and Brighton Criterion in order to diagnosis of joint hypermobility in children. Journal of Education, Health and Sport. 2018;8(5):133–148

[9] Przymuszała A, Roszak M, Kulik O, et al Generalised joint hypermobility as a symptom of chosen diseases and syndromes. Journal of Education, Health and Sport. 2018;8(4):246–255

[10] Czaprowski D, Kotwicki T, Stoliński Ł. Assessment of Joint Laxity in Children and Adolescent–a Review of Methods. Ortopedia, Traumatologia, Rehabilitacja. 2012;5(6),14: 407–420 10.5604/15093492.1016368 23208932

[11] Murray KJ. Hypermobility disorders in children and adolescents. Best Practice & Research Clinical Rheumatology. 2006;20:329–351 16546060 10.1016/j.berh.2005.12.003

[12] Czaprowski D, Kotwicki T, Pawłowska P, Stoliński Ł. Joint hypermobility in children with idiopathic scoliosis: SOSORT award 2011 winner. Scoliosis. 2011;6:22, 10.1186/1748-7161-6-22 21981906 PMC3204294

[13] Adib N, Davies K, Grahame R, Woo P, Murray KJ. Joint hypermobility syndrome in childhood. A not so benign multisystem disorder? Rheumatology. 2005;44:744–750 10.1093/rheumatology/keh557 15728418

[14] Hanewinkel-van Kleef YB, Helders PJM, Takken T, Engelbert RH. Motor performance in children with generalized joint hypermobility: The influence of muscle strength and exercise capacity. Pediatric Physical Therapy. 2009;21(2):194–200 10.1097/PEP.0b013e3181a3ac5f 19440129

[15] Smits-Engelsman B, Klerks M, Kirby A. Beighton Score: A Valid Measure for Generalized Joint Hypermobility in Children. Journal of Pediatric Orthopedics. 2011;158:119–123 10.1016/j.jpeds.2010.07.021 20850761

[16] Scheper MC, Engelbert RHH, Rameckers EAA, Verbunt J, Remvig L, Juul-Kristensen B. Children with Generalized Joint Hypermobility and Musculoskeletal Complaints: State of the Art on Diagnostics, Clinical Characteristics, and Treatment. BioMed Research International. 2013; ID 121054, 1–13 10.1155/2013/121054 PMC3736514 23971021

[17] Engelbert RH, Bank RA, Sakkers RJ, Helders PJ, Beemer FA, Uiterwaal CS. Pediatric generalised joint hypermobility with and without musculoskeletal complaints: a localized or systematic disorder? Pediatrics. 2003;111:e248–254 10.1542/peds.111.3.e248 12612280

[18] Keer R, Grahame R. Hypermobility Syndrome. Recognition and Management for Physiotherapists. Physical Therapy in Sport. 2004;5(1):51

[19] Kerr A, Macmillan CE, Uttley WS, Luqmani RA: Physiotherapy for Children with Hypermobility Syndrome. Physiotherapy. 2000;86(6),313–317

[20] Czaprowski D, Kędra A, Pawłowska P, Kolwicz-Gańko A, Leszczewska J, Tyrakowski M. The Examination of the Musculoskeletal System Based Only on the Evaluation of Pelvic-Hip Complex Muscle and Trunk Flexibility May Lead to Failure to Screen Children for Generalized Joint Hypermobility. PLoS ONE 2015;10(3):e0121360 10.1371/journal.pone.0121360 25786251 PMC4364744

[21] Kabiri LS, Hernandez DC, Mitchell K. Reliability, Validity, and Diagnostic Value of a Pediatric Bioelectrical Impedance Analysis Scale. Childhood Obesity. 2015;11(5):650–655 10.1089/chi.2014.0156 26332367

[22] Alvares JBAR, Rodrigues R, Franke RA, Silva BGC, Pinto RS, Vaz MA, Baroni BM. Inter-machine reliability of the Biodex and Cybex isokinetic dynamometers for knee flexor/extensor isometric, concentric and eccentric tests. Physical Therapy in Sport. 2015;16:59–65 10.1016/j.ptsp.2014.04.004 24913915

[23] Tsiros MD, Grimshaw PN, Schield AJ, Buckley JD. Test-retest reliability of the Biodex System 4 Isokinetic Dynamometer for knee strength assessment in paediatric populations. Journal Of Allied Health. 2011;40(3):115–119 21927776

[24] Slinde F, Suber C, Suber L, Edwen CE, Svantesson U. Test-Retest Reliability of Three Different Countermovement Jumping Tests. Journal of Strength and Conditioning Research. 2008;22(2):640–644 10.1519/JSC.0b013e3181660475 18550985

[25] Cormack SJ, Newton RU, McGuigan MR, Doyle TLA. Reliability of Measures Obtained During Single and Repeated Countermovement Jumps. International Journal of Sports Physiology and Performance. 2008;3:131–144 10.1123/ijspp.3.2.131 19208922

[26] Hakim A, Malfait F, De Paepe A. The heritable disorders of connective Tissue: epidemiology, nosology and clinical features. In: Hakim A, Keer R, Grahame R, editors. Hypermobility, fibromyalgia and chronic pain. Edinburgh. Elsevier 2010:6

[27] Juul-Kristensen B, Schmedling K, Rombaut L, Lund H, Engelbert RHH. Measurement properties of clinical assessment methods for classifying generalized joint hypermobility–A systematic review. American Journal of Medical Genetics Seminars in Medical Genetics. 2017;175(1):116–147 10.1002/ajmg.c.31540 28306223

[28] Prins M, Hawkesworth S, Wright A et al Use of bioelectrical impedance analysis to assess body composition in rural Gambian children. European Journal of Clinical Nutrition. 2008; 62:1065–1074 10.1038/sj.ejcn.1602830 17622262

[29] Boyd B.S: Measurement properties of a hand-held inclinometer during straight leg raise neurodynamic testing. Physiotherapy, 2012;98:174–179 10.1016/j.physio.2011.04.352 22507369

[30] Trzaskoma Z, Ilnicka L, Wiszomirska I, Wit A, Wychowański M. Laterality versus jumping performance in men and women. Acta of Bioengineering and Biomechanics. 2015;17(1): 103–110 25951855

[31] Rombaut L, Malfait F, De Wandele I, Taes Y, Thijs Y, De Paepe A, et al Muscle Mass, Muscle Strength, Functional Performance, and Physical Impairment in Women With the Hypermobility Type of Ehlers-Danlos Syndrome. Arthritis Care & Research. 2012;64(10): 1584–1592 22556148 10.1002/acr.21726

[32] Drouin JM, Valovich-mcLeod TC, Shultz SJ, Gansneder BM, Perrin DH. Reliability and validity of the Biodex System 3 Pro isokinetic dynamometer velocity, torque and position measurements. European Journal of Applied Physiology. 2004;91(1):22–29 10.1007/s00421-003-0933-0 14508689

[33] Wang Y.C., Bohannon R.W., Magasi S.R., et al Testing of knee extension muscle strength: A comparison of two portable alternatives for the NIH toolbox study. Isokinetics and Exercise Science. 2011;19(3):163–168

[34] Juul-Kristensen B, Hansen H, Simonsen EB, Alkjear T, Kristensen JH, Jensen BR, et al Knee function in 10-year-old children and adults with Generalised Joint Hypermobility. The Knee. 2012;19:773–778 10.1016/j.knee.2012.02.002 22417629

[35] Sahin N, Baskent A, Ugurlu H, Berker E. Isokinetic evaluation of knee extensor/flexor muscle strength in patients with hypermobility syndrome. Rheumatology International. 2008;28:643–648 10.1007/s00296-007-0493-4 18043921

[36] Van Meeteren J, Roebroeck ME, Stam HJ. Test-Retest—Reliability in Isokinetic Muscle Strength Measurements of the Shoulder. Journal of Rehabilitation Medicine. 2002;34:91–95 10.1080/165019702753557890 12019586

[37] Kaminska E, Piontek T, Wiernicka M, Cywinska-Wasilewska G, Lewandowski J, Lochynski D. Differences in Isokinetic Strength of the Knee Extensors and Flexors in Men With Isolated and Combined Cruciate-Ligament Knee Injury. Journal of Sport Rehabilitation. Human Kinetics. 2015;24:268–277 10.1123/jsr.2014-0157 25158093

[38] Kelly SB, Alvar BA, Black LE, Dodd DJ, Carothers KF, Brown LE. Vibration vs. Cycle Ergometry on Isokinetic Dynamometry. The Journal of Strength and Conditioning Research. 2010; 24(11):3140–3143 10.1519/JSC.0b013e3181f9278f 20940645

[39] Markovic S, Mirkov DM, Nedeljkovic A, Jaric S. Body size and countermovement depth confound relationship between muscle power output and jumping performance. Human Movement Science. 2014;33:203–210 10.1016/j.humov.2013.11.004 24280557 PMC3943730

[40] Holden S, Boreham C, Doherty C, Wang D, Delahunt E. Clinical assessment of countermovement jump landing kinematics in early adolescence: Sex differences and normative values. Clinical Biomechanics. 2015;30:469–474 10.1016/j.clinbiomech.2015.03.008 25836628

[41] Engelbert RH, van Bergen M, Henneken T, Helders PJ, Takken T. Exercise tolerance in children and adolescents with musculoskeletal pain in joint hypermobility and joint hypomobility syndrome. Pediatrics. 2006;118(3):e690–696 10.1542/peds.2005-2219 16950961

[42] Sohrbeck-Nøhr O, Kristensen JH, Boyle E, Remvig L, Juul-Kristensen B. Generalized joint hypermobility in childhood is a possible risk for the development of joint pain in adolescence: a cohort study. BMC Pediatrics. 2014;14:302–400 10.1186/s12887-014-0302-7 25492414 PMC4305244

[43] Jensen BR, Olesen AT, Pedersen MT, et al Effect of generalized joint hypermobility on knee function and muscle activation in children and adults. Muscle & Nerve. 2013;48:762–769 24037762 10.1002/mus.23802

[44] Junge T, Wedderkopp N, Thorlund JB, Søgaard K, Juul-Kristensen B. Altered knee joint neuromuscular control during landing from a jump in 10–15 year old children with Generalised Joint Hypermobility. A substudy of the CHAMPS-study Denmark. Journal of Electromyography & Kinesiology. 2015;25(3):501–508 25801907 10.1016/j.jelekin.2015.02.011

[45] Jindal P, Narayan A, Ganesan S, MacDermid JC. Muscle strength differences in healthy young adults with and without generalized joint hypermobility. BMC Sports Sciences, Medicine and Rehabilitation. 2016;8–12, 10.1186/s13102-016-0037-x PMC4845357 27119015

[46] Liaghat B, Juul-Kristensen B, Frydendal T, Marie Larsen C, Søgaard K, Ilkka Tapio Salo A. Competitive swimmers with hypermobility have strength and fatigue deficits in shoulder medial rotation. Journal of Electromyography & Kinesiology. 2018;39,1–7 29353138 10.1016/j.jelekin.2018.01.003

[47] Mebes C; Amstutz A, Luder G, et al Isometric rate of force development, maximum voluntary contraction, and balance in women with and without joint hypermobility. Arthritis And Rheumatism. 2008;15;59(11):1665–1669 10.1002/art.24196 18975361

[48] Massy-Westropp N, Toubia Ch. Hypermobility as measured by the Beighton hypermobility test is not predictive of hand grip strength in young adults. Journal of Musculoskeletal Research. 2013;16(1):1350006–1–5

[49] Yazgan P, Duymaz T. Grip strength in joint hypermobility syndrome. Annals of the Rheumatic Diseases. 2015;74:1220 10.1136/annrheumdis-2015-eular.6201

[50] Fayed N, Cieza A, Edmond BJ. Linking health and health-related information to the ICF: a systematic review of the literature from 2001 to 2008. Disability and Rehabilitation. 2011;33(21–22):1941–1951 10.3109/09638288.2011.553704 21303198

[51] Fatoye FA, Palmer S, Macmillan F, Rowe PJ, van der Linden ML. Proprioception and muscle torque deficits in children with hypermobility syndrome. Rheumatology. 2009;48:152–157 10.1093/rheumatology/ken435 19088133

[52] Scheper M, de Vries J, Beelen A, de Vos R, Nollet F, Engelbert R. Generalized joint hypermobility, muscle strength and physical function in healthy adolescents and young adults. Current Rheumatology Reviews. 2015;10(2):117–125 10.2174/1573397111666150120112925 25599680

[53] Remvig L, Kümmel C, Kristensen JH, Boas G, Juul-Kristensen B. Prevalence of generalized joint hypermobility, arthralgia and motor competence in 10-year-old school children. International Musculoskeletal Medicine. 2011;33(4):137–145

[54] Loturco I, Nakamura FY, Artioli GG, et al Strength and power qualities are highly associated with punching impact in elite amateur boxers. Journal of Strength and Conditioning Research. 2016;30(1):109–116 10.1519/JSC.0000000000001075 26110348

