# Correction: Muscle strength, muscle power and body composition in college-aged young women and men with Generalized Joint Hypermobility

**DOI:** 10.1371/journal.pone.0252265

**Published:** 2021-05-20

**Authors:** Paulina Ewertowska, Zbigniew Trzaskoma, Dominik Sitarski, Bartłomiej Gromuł, Ireneusz Haponiuk, Dariusz Czaprowski

There are errors in the author affiliations. The correct affiliations are as follows:

Paulina Ewertowska^1^, Zbigniew Trzaskoma^2^, Dominik Sitarski^3^, Bartłomiej Gromuł^3^, Ireneusz Haponiuk^1^, Dariusz Czaprowski^4^

**1** Department of Physical Culture, Gdansk University of Physical Education and Sport, Gdansk, Poland, **2** Faculty of Rehabilitation, Józef Piłsudski University of Physical Education, Warsaw, Poland, **3** Department of Health Sciences, Olsztyn University, Olsztyn, Poland, **4** Physiotherapy Unit, Department of Health Sciences, Poznan University of Medical Sciences, Poznan, Poland.
