# Supplementary material for: Muscle strength, muscle power and body composition in college-aged young women and men with Generalized Joint Hypermobility
Source: PLoS One. 2020 Jul 29;15(7):e0236266. doi: 10.1371/journal.pone.0236266 (PMC7390387; doi:10.1371/journal.pone.0236266)
Supplement: S1 Table — (DOC) [file pone.0236266.s001.doc]

| Table 1. Characteristics of women and men with GJH and from the CG | | | | | | |
| --- | --- | --- | --- | --- | --- | --- |
|  | Females n=53 | | | Males n=34 | | |
|  | GJH  n=25 | CG  n=28 | p value | GJH  n=15 | CG  n=19 | p value |
|  | Me (IQR) | Me (IQR) |  | Me (IQR) | Me (IQR) |  |
| Age (years) | 21.0 (20.0-23.0) | 21.0 (19.0-22.0) | .68 | 21.0 (20.0-21.0) | 21.0 (20.0-22.5) | .06 |
| Weight (kg) | 60.1 (54.8-67.9) | 61.2 (54.5-70.1) | .55 | 80.0 (75.0-87.0) | 78.0 (72.9-82.7) | .42 |
| Height (m) | 1.7 (1.6-1.7) | 1.7 (1.6-1.7) | .13 | 1.8 (1.8-1.9) | 1.8 (1.8-1.9) | .49 |
| BMI (kg/m^2^) | 21.9 (19.8-24.6) | 21.8 (19.7-25.8) | .72 | 24.6 (22.4-25.8) | 24.1 (20.9-25.3) | .31 |
| Physical activity  (h/week) | 3.0 (2.0-6.0) | 4.0 (2.0-6.0) | .27 | 6.0 (5.0-7.5) | 6.5 (4.0-11.5) | .24 |
| Abbreviations: GJH – Generalized Joint Hypermobility, CG – Control Group, Me – Median, IQR – Interquartile Range. | | | | | | |
