# Supplementary material for: Muscle strength, muscle power and body composition in college-aged young women and men with Generalized Joint Hypermobility
Source: PLoS One. 2020 Jul 29;15(7):e0236266. doi: 10.1371/journal.pone.0236266 (PMC7390387; doi:10.1371/journal.pone.0236266)
Supplement: S2 Table — (DOC) [file pone.0236266.s002.doc]

| Table 2. The comparison of body compositions between females and males with and without Generalized Joint Hypermobility | | | | | | |
| --- | --- | --- | --- | --- | --- | --- |
|  | Females n=53 | | | Males n=34 | | |
|  | GJH  n=25 | CG  n=28 | p value | GJH  n=15 | CG  n=19 | p value |
|  | Mean (SD) | Mean (SD) |  | Mean (SD) | Mean (SD) |  |
| Fat mass (%) | 28.4 (5.8) | 29.2 (7.1) | .68 | 14.2 (3.6) | 14.6 (5.7) | .82 |
| Fat mass (kg) | 17.9 (6.0) | 19.8 (8.7) | .41 | 11.6 (3.8) | 11.5 (4.7) | .98 |
| Fat-free mass (kg) | 43.6 (3.2) | 45.3 (5.9) | .56 | 69.2 (7.0) | 66.7 (6.1) | .37 |
| Total body water (kg) | 31.9 (2.4) | 33.1 (4.3) | .57 | 50.7 (5.1) | 48.8 (4.5) | .36 |
| Abbreviations: GJH – Generalized Joint Hypermobility, CG – Control Group, SD – Standard Deviation. | | | | | | |
