# Supplementary material for: Muscle strength, muscle power and body composition in college-aged young women and men with Generalized Joint Hypermobility
Source: PLoS One. 2020 Jul 29;15(7):e0236266. doi: 10.1371/journal.pone.0236266 (PMC7390387; doi:10.1371/journal.pone.0236266)
Supplement: S3 Table — (DOC) [file pone.0236266.s003.doc]

| Table 3. The comparison of muscle flexibility tests between females and males with and without Generalized Joint Hypermobility | | | | | | |
| --- | --- | --- | --- | --- | --- | --- |
|  | Females n=53 | | | Males n=34 | | |
|  | GJH  n=25 | CG  n=28 | p value | GJH  n=15 | CG  n=19 | p value |
| Lower extremity | Mean (SD) | Mean (SD) |  | Mean (SD) | Mean (SD) |  |
| Dominant |  |  |  |  |  |  |
| SLR (°) | 65.5 (9.7) | 65.8 (8.9) | .91 | 57.9 (12.9) | 56.7 (9.7) | .81 |
| PA (°) | 67.8 (12.0) | 66.0 (8.8) | .55 | 64.1 (13.7) | 61.2 (9.2) | .81 |
| O-JHF (°) | 17.5 (8.0) | 16.1 (7.5) | .46 | 11.1 (15.3) | 4.2 (16.4) | .27 |
| T-JHF (°) | 78.4 (7.8) | 78.1 (9.2) | .88 | 68.6 (9.7) | 73.9 (9.1) | .16 |
| Non-dominant |  |  |  |  |  |  |
| SLR (°) | 63.9 (9.6) | 63.8 (10.5) | .96 | 55.6 (12.9) | 57.1 (9.0) | .74 |
| PA (°) | 67.9 (10.9) | 66.6 (8.4) | .61 | 63.6 (15.3) | 58.7 (11.7) | .54 |
| O-JHF (°) | 18.9 (9.6) | 16.7 (8.9) | .26 | 13.1 (13.8) | 5.6 (14.5) | .18 |
| T-JHF (°) | 78.5 (8.1) | 77.3 (8.7) | .61 | 65.9 (14.1) | 72.5 (10.4) | .18 |
| Abbreviations: GJH – Generalized Joint Hypermobility, CG – Control Group, SD – Standard Deviation, SLR – Straight Leg Raise test, PA – Popliteal Angle test, O-JHF – One-Joint Hip Flexors test; T-JHF – Two-Joint Hip Flexors test. | | | | | | |
