# Supplementary material for: Muscle strength, muscle power and body composition in college-aged young women and men with Generalized Joint Hypermobility
Source: PLoS One. 2020 Jul 29;15(7):e0236266. doi: 10.1371/journal.pone.0236266 (PMC7390387; doi:10.1371/journal.pone.0236266)
Supplement: S4 Table — (DOC) [file pone.0236266.s004.doc]

| Table 4. The comparison of peak torque under isokinetic conditions obtained by females and males with and without Generalized Joint Hypermobility | | | | | | | |
| --- | --- | --- | --- | --- | --- | --- | --- |
| Peak torque/  Body weight  (Nm/kg) | | Females n=53 | | | Males n=34 | | |
|  |  | GJH  N=25 | CG  n=28 | p value | GJH  n=15 | CG  n=19 | p value |
| Lower extremity | | Mean (SD) | Mean (SD) |  | Mean (SD) | Mean (SD) |  |
| Dominant | |  | | | | | |
| Flexion | 60°/s | 0.87 (0.19) | 0.90 (0.23) | .57 | 1.17 (0.24) | 1.28 (0.32) | .39 |
|  | 180°/s | 0.64 (0.18) | 0.63 (0.21) | .97 | 0.82 (0.19) | 0.91 (0.23) | .31 |
| Extension | 60°/s | 2.13 (0.31) | 2.05 (0.43) | .53 | 2.55 (0.39) | 2.63 (0.37) | .54 |
|  | 180°/s | 1.29 (0.21) | 1.29 (0.33) | .46 | 1.55 (0.35) | 1.68 (0.15) | .23 |
| F/E (-) | 60°/s | 0.41 (0.07) | 0.44 (0.07) | .29 | 0.47 (0.13) | 0.48 (0.10) | .74 |
|  | 180°/s | 0.50 (0.12) | 0.48 (0.10) | .57 | 0.54 (0.09) | 0.57 (0.13) | .39 |
| Non-dominant | |  | | | | | |
| Flexion | 60°/s | 0.85 (0.17) | 0.86 (0.21) | .87 | 1.07 (0.24) | 1.21 (0.30) | .12 |
|  | 180°/s | 0.66 (0.15) | 0.60 (0.24) | .16 | 0.73 (0.18) | 0.93 (0.23) | **.04*** |
| Extension | 60°/s | 2.00 (0.28) | 2.05 (0.44) | .79 | 2.15 (0.63) | 2.59 (0.42) | **.02*** |
|  | 180°/s | 1.23 (0.18) | 1.25 (0.33) | .36 | 1.39 (0.37) | 1.65 (0.33) | **.03*** |
| F/E (-) | 60°/s | 0.43 (0.09) | 0.42 (0.06) | .74 | 0.52 (0.11) | 0.47 (0.09) | .16 |
|  | 180°/s | 0.55 (0.11) | 0.48 (0.12) | **.04*** | 0.54 (0.07) | 0.57 (0.11) | .40 |
| Abbreviations: GJH – Generalized Joint Hypermobility, CG – Control Group, SD – Standard Deviation, F/E – Flexion/Extension ratio, * Statistically significant differences. | | | | | | | |
