# Supplementary material for: Muscle strength, muscle power and body composition in college-aged young women and men with Generalized Joint Hypermobility
Source: PLoS One. 2020 Jul 29;15(7):e0236266. doi: 10.1371/journal.pone.0236266 (PMC7390387; doi:10.1371/journal.pone.0236266)
Supplement: S5 Table — (DOC) [file pone.0236266.s005.doc]

| Table 5. The comparison of peak torque under isometric conditions obtained by females and males with and without Generalized Joint Hypermobility | | | | | | |
| --- | --- | --- | --- | --- | --- | --- |
| Peak torque/  Body weight  (Nm/kg) | Females n=53 | | | Males n=34 | | |
|  | GJH  n=25 | CG  n=28 | p value | GJH  n=15 | CG  n=19 | p value |
| Lower extremity | Mean (SD) | Mean (SD) |  | Mean (SD) | Mean (SD) |  |
| Dominant |  | | | | | |
| Flexion 30° | 1.30 (0.22) | 1.38 (0.24) | .25 | 1.85 (0.20) | 1.93 (0.26) | .36 |
| Extension 70° | 2.28 (0.41) | 2.32 (0.55) | .76 | 3.02 (0.47) | 3.02 (0.48) | .98 |
| F/E (-) | 0.58 (0.08) | 0.61 (0.11) | .25 | 0.62 (0.08) | 0.65 (0.07) | .37 |
| Non-dominant |  | | | | | |
| Flexion 30° | 1.20 (0.20) | 1.28 (0.22) | .19 | 1.64 (0.26) | 1.82 (0.29) | .08 |
| Extension 70° | 2.11 (0.46) | 2.32 (0.48) | .16 | 2.69 (0.79) | 2.82 (0.38) | .09 |
| F/E (-) | 0.59 (0.13) | 0.56 (0.11) | .56 | 0.65 (0.18) | 0.65 (0.08) | .28 |
| Abbreviations: GJH – Generalized Joint Hypermobility, CG – Control Group, SD – Standard Deviation, F/E – Flexion/Extension ratio. | | | | | | |
