# Supplementary material for: Muscle strength, muscle power and body composition in college-aged young women and men with Generalized Joint Hypermobility
Source: PLoS One. 2020 Jul 29;15(7):e0236266. doi: 10.1371/journal.pone.0236266 (PMC7390387; doi:10.1371/journal.pone.0236266)
Supplement: S6 Table — (DOC) [file pone.0236266.s006.doc]

| Table 6. The comparison of maximal muscle power and jumping ability obtained by females and males with and without Generalized Joint Hypermobility | | | | | | |
| --- | --- | --- | --- | --- | --- | --- |
|  | Females n=53 | | | Males n=34 | | |
|  | GJH  n=25 | CG  n=28 | p value | GJH  N=15 | CG  n=19 | p value |
|  | Mean (SD) | Mean (SD) |  | Mean (SD) | Mean (SD) |  |
| Maximal Power (W/kg) | 16.6 (3.8) | 17.1 (4.2) | .66 | 23.1 (3.6) | 22.8 (4.7) | .85 |
| Height of jump (cm) | 25.3 (2.9) | 25.9 (3.9) | .52 | 34.3 (5.8) | 35.3 (5.4) | .64 |
| Abbreviations: GJH – Generalized Joint Hypermobility, CG – Control Group, SD – Standard Deviation. | | | | | | |
